# An Unusual Polypoid Septal Mucosal Melanoma: Overview and Diagnostic Pitfalls

**DOI:** 10.7759/cureus.20819

**Published:** 2021-12-30

**Authors:** Spyridon Lygeros, Alexandra Danielidi, Vasiliki Tzelepi, Katerina Grafanaki

**Affiliations:** 1 Department of Otorhinolaryngology-Head and Neck Surgery, School of Medicine, University of Patras, Patras, GRC; 2 Department of Dermatology, School of Medicine, University of Tübingen, Tübingen, DEU; 3 Department of Pathology, School of Medicine, University of Patras, Patras, GRC; 4 Department of Dermatology, School of Medicine, University of Patras, Patras, GRC

**Keywords:** sinonasal mass, sinonasal tumor pathology, nasal mucosa, nasal septum, mucosal malignant melanoma

## Abstract

Sinonasal mucosa is an area of high melanocyte density compared to other mucosa-lined sites. Sinonasal mucosal melanomas (SNMM) most commonly arise from the nasal cavity and the paranasal sinuses. Due to their obscure anatomic location and lack of early symptomatology, SNMM are often diagnosed in an advanced stage. The majority of patients who present with symptoms complain of unilateral nasal dysfunction, such as obstruction and epistaxis.

We hereby report a case of an 86-year-old female, who presented with a three-year history of progressive right-sided nasal obstruction and recurrent epistaxis. Posterior rhinoscopy and endoscopy revealed a polypoid, fleshy lesion whose coloration varied from mildly pigmented to amelanotic. Inverted sinonasal papilloma was included in the differential diagnosis due to MRI findings. Post-resection histopathology indicated a mucosal melanoma. Typically, amelanotic lesions are rare, more difficult to diagnose and associated with worse prognosis due to both their aggressiveness and delayed diagnosis.

## Introduction

Mucosal melanomas (MM) are rare malignancies that account for 1-4% of all melanomas and present a female predilection (1.2 vs 1 per million). The sinonasal mucosa is an area of high melanocyte density compared to other mucosal surfaces and sinonasal mucosal melanomas (SNMM) most commonly arise in the nasal cavity rather than the paranasal sinuses [1]. Due to their anatomic origin and the non-specific or absent early symptomatology, SNMM are usually diagnosed in an advanced stage. Patients who present with symptoms typically complain of unilateral nasal obstruction and recurrent epistaxis, while other symptoms may include rhinorrhea, hyposmia and frontal headache [2].

We report the case of an 86-year-old patient with septal MM presenting with chronic unilateral nasal dysfunction. 

## Case presentation

An 86-year-old female presented to the ENT Department due to a three-year history of progressively worsening right-sided nasal obstruction and recurrent episodes of epistaxis. Anterior rhinoscopy and fiberoptic nasoendoscopy revealed a large mass with macroscopically heterogenous pigmentation, ranging from completely amelanotic to mildly pigmented areas. The mass was totally obstructing the right nasal cavity and extended to the nasopharynx. Lymphadenopathy was not detected. The patient had been receiving medical therapy for hypertension and osteoporosis and had no history of smoking, alcohol abuse or exposure to chemicals, such as formaldehyde.

**Figure 1:**
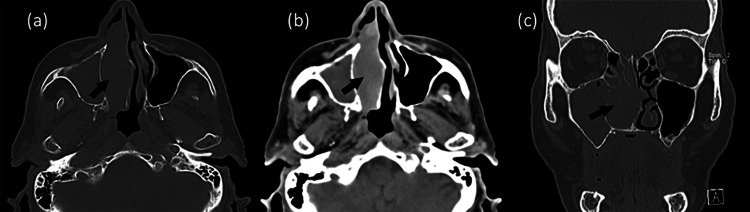
Computed Tomography (CT) of the nasal sinuses. Large mass deriving from the nasal septum, causing total obstruction of the right nasal cavity, chronic inflammation of the right maxillary sinus and right ethmoid cells and deviation of the nasal septum. Axial view: (a) and (b). Coronal view (c).

**Figure 2:**
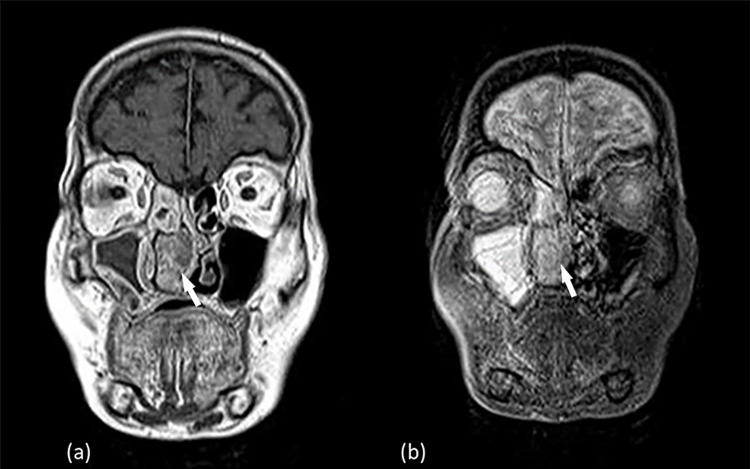
MRI coronal section. In the T1-weighted sequence the mass appears heterogeneous with low intensity signal (a). In the T2-weighted sequence is observed the heterogeneous mass with intermediate to high signal intensity (b).

**Figure 3:**
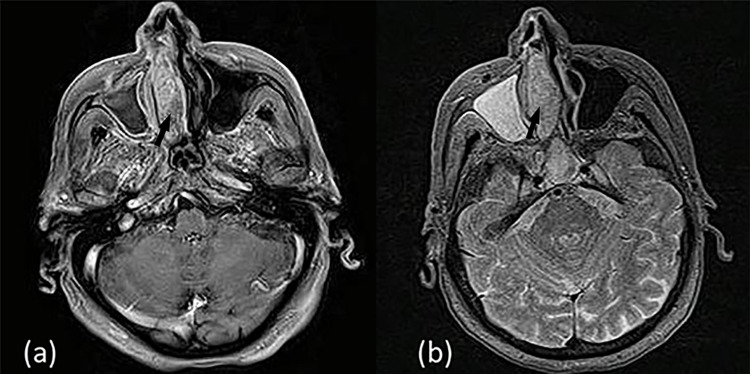
MRI axial section. (a) T1-weighted sequence with gadolinium enhancement (b) T2-weighted sequence.

Computed tomography (CT) of the facial sinuses demonstrated a large mass of the right nasal cavity, measuring 4.5 x 2.5 x 1.8 cm along its largest axis, originating from the nasal septum and totally obstructing the right nasal cavity. The mass expanded in the nasal cavity and caused bone erosion to the right inferior turbinates (Figure 1). In magnetic resonance imaging (MRI), the mass had low signal intensity on T1- and heterogeneous intermediate to high signal intensity on T2-weighted sequences (Figures 2, 3). Findings of chronic inflammation were present in the right maxillary sinus, right ethmoidal cells and right sphenoid sinus, attributable to the chronicity of obstruction. The differential diagnosis included sinonasal inverted papilloma (SNIP), a common benign sinonasal epithelial neoplasm, with high recurrence rate and possible association with squamous cell carcinoma (SCC).

**Figure 4:**
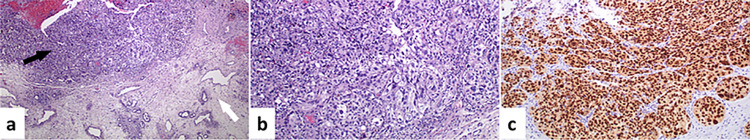
Histopathology images. (a) A malignant neoplasm is seen on the surface of the nasal mucosa (black arrow). Note non-neoplastic nasal glands at the lower part of the image (white arrow)(original magnification X40). (b) Neoplastic cells are arranged in a nested-nodular pattern, have pleomorphic nuclei with distinct nucleoli and abundant cytoplasm (original magnification X100). (c) Diffuse intense expression of SOX10 is seen in the neoplastic cells (original magnification X100).

An extended biopsy was performed along with functional endoscopic sinus surgery (FESS) to restore sinus ventilation and normal function. Microscopy revealed a malignant neoplasm that exhibited immunohistochemical positivity against SOX10, Melan-A, HMB45, MiTF and CD117/C-KIT, whereas the neoplastic cells were negative for CKAE1/AE3. Both histologic and immunohistochemical findings were consistent with a malignant mucosal melanoma (Figure 4). BRAF mutation was not detected on polymerase chain reaction (PCR) molecular testing.

To assess the stage of the disease, a full body CT and PET-CT scans were performed, without evident lymph node involvement or distant metastases. The tumor was classified as Stage III (T3N0M0) according to the American Joint Committee on Cancer (8th edition) staging system for MM [3]. The patient was referred to the Oncology Department and postoperative immunotherapy with pembrolizumab, a programmed death- 1 (PD-1) inhibitor was initiated, aiming to eradicate any residual neoplastic disease. Four months postoperatively, the patient is being followed up on an outpatient basis by Oncology, with no further complications reported from treatment to date.

## Discussion

Mucosal melanomas are rare malignancies that constitute less than 4% of all head and neck cancers, with a higher incidence in women, usually between 60 and 80 years of age [2]. 

The sinonasal mucosa is characterized by high melanocyte density and is the most common location for exposure to carcinogens like formaldehyde which was for decades considered to be a risk factor for MM development. However, many studies have failed to confirm this hypothesis [4]. Sunlight does not seem to predispose to MM unlike cutaneous melanoma (CM), as they occur in sun-shielded surfaces. This is also reflected on the type of mutations mucosal melanoma tumors tend to accumulate (over the course of their progression). UV-radiation-derived mutations of the BRAF gene are only detected in 0-3% of all MM, whereas rare c-KIT somatic mutations are encountered more frequently than in CM, as in our case. The immunohistochemical identity of our patient’s tumor is in accordance with its mucosal origin [4,5].

The majority of sinonasal mucosal melanomas (SNMM) arise from the nasal cavity (80% of cases) while the remainder originates from the paranasal sinuses (20% of cases). Malignancy usually derives from the lateral nasal wall, followed by the nasal septum, maxillary sinus, and ethmoid sinus. Except for the typical complaint of unilateral nasal dysfunction, facial pain, V2 hypoesthesia and ophthalmologic signs (epiphora, vision loss, exophthalmos, ptosis) may rarely be observed and are associated with advanced-stage disease [6].

In the present case, the color of the MM varied from minimally pigmented to amelanotic. Interestingly, amelanotic lesions, in spite of their rarity, are linked to poor prognosis, due to their aggressive behavior, as well as their typically impeded identification and management. Previous cases of masses with different degrees of pigmentation within the same mass as well as satellite lesions surrounding the main tumor have been reported [7].

MRI with contrast agent is regarded as the gold standard for diagnosing unilateral sinonasal pathology as it is capable of distinguishing solid lesions from inflammation. The hallmarks of radiological findings differentiating malignant sinonasal tumors from SNIP are bone erosion on CT, involvement of adjacent structures, and intermediate signal intensity on T2-weighted MRI sequences. Both SNIP and SNIP-SCC have different degrees of enhancement on MRI. Especially in dynamic MRI enhancement studies, the inflow and outflow velocities of the contrast agents differ due to variability of blood vessel constriction, which leads to differences in the dynamic enhancement curves. This difference is an important index for the diagnosis and differentiation of malignant transformation. Both malignant and non-malignant inverted papilloma can cause bone hyperplasia and calcification [8]. MMs, on the other hand, are depicted as lesions of high signal intensity on T1- and hypo signal intensity on T2-weighted images, mainly due to the high melanin content [9]. Indeed, radiological findings depend on the amount of the tumor melanin and the degree of hemorrhage and can often vary. However, variations in signal intensity interpretation set an additional barrier to the diagnosis of this rare malignancy, as MMs can be misdiagnosed based solely on MRI findings [9]. Highly melanotic tumors (>10% melanin-containing cells) are associated with T1-hyperintensity, while tumors with <10% melanin-containing cells produce T1-isointensity [10] and increased T1 signal is attributed to intratumoral hemorrhage. Finally, it has been reported that atypical findings in MRI such as low signal intensity on T1- and a heterogeneous intermediate to high signal intensity on T2-weighted images can be associated with worse prognosis and poor outcome [9-13].

Accordingly, our patient’s MRI demonstrated low signal intensity of the lesion on T1- and heterogeneous intermediate to high signal intensity on T2-weighted sequences, a finding which posed inverted papilloma high in the differential diagnosis [8]. In a similar case reported by Kim et al. [9], MRI findings, as well as the macroscopic appearance of the lesion, were also misleading, rendering histopathologic examination indispensable for accurate diagnosis and management.

Regarding the treatment modalities available, surgical wide excision still remains the first line of treatment for this type of melanomas. Meanwhile therapeutic strategies include controlling local residual disease and preventing distant metastasis. Selective neck dissection and sentinel node biopsy (SLNB) in lymph node negative patients are controversial, whereas the role of post-surgical radiotherapy as adjuvant needs further elucidation [14].

In our case, due to the long course of our patient’s disease and the lack of severe functional impairment, an extended excisional biopsy was endoscopically performed, as FESS produces similar oncologic outcomes and carries smaller risk when compared to traditional open surgical approaches [1]. Clear surgical margins, although reportedly leading to better outcomes when achieved, were rather challenging to obtain in our case, given the tumor’s relatively large size and the anatomic complexities of the region. SLNB was not taken into consideration due to the negative imaging results on nodal involvement, the technical feasibility and the patient’s age.

Immune checkpoint inhibitors (ICIs) have been an advance in the management of melanoma; however, mechanisms of response and resistance remain poorly understood. Systemic adjuvant therapy with ICIs or targeted therapies have begun to demonstrate a benefit on mortality rates after complete resection of high-risk melanoma (stages III and IV), whereas outcomes remain poor for advanced (metastatic) disease [3]. Combination immunotherapy with PD-1 and cytotoxic T lymphocyte-associated antigen (CTLA-4) is the first-line treatment for MM. However, in patients unfit for combination immunotherapy, nivolumab or pembrolizumab monotherapy is opted. In the presence of certain mutations [4], BRAF or c-KIT targeted therapy is indicated in urgently desired symptomatic benefit or in case of failure of immunotherapy [1,3,14].

Despite these advances, patients with SNMM show limited benefit from current therapeutic strategies and complete excision of the tumor is widely regarded as the sole, proven efficient treatment modality for SNMM [1,13]. Based on the patient’s age and clinical profile, our multidisciplinary team concluded that adjuvant therapy with pembrolizumab, a PD-1 inhibitor with an established efficacy in MMs, would be the optimum treatment plan. Programmed cell death ligand 1 (PD-L1) is a potential predictive marker for response and outcome after treatment with PD-1. PD-1 is expressed in about one-quarter of all MMs, exhibiting a similar therapeutic response as in CM. Immunotherapy with PD-1 inhibitors has been found to increase the overall response rate in both PD-L1 positive (PD-L1 expression >= 5%) and negative MMs [2].

## Conclusions

MM is a very rare and aggressive neoplasm that requires vigilance. In the present case, adjuvant immunotherapy was implemented post-surgically for an optimal outcome aiming to eradicate any residual MM, as given the bulk of the tumor, clear surgical margins were impossible to obtain. The slow growth rate of this mildly colored tumor, the complexity of the nasal and septal anatomy and limited accessibility, as well as the longstanding non-characteristic symptomatology, highlight that high clinical suspicion is required, especially in the case of hypomelanotic/amelanotic MM. Moreover, while MRI is the gold standard of imaging for diagnosis, it may occasionally lead to diagnostic pitfalls. Lastly, as with this case, therapeutic options may be limited due to the patient’s age and clinical profile, rendering SNMM survival and prognosis improvement rather problematic.
